# Study of the Behavior of a Bell-Shaped Colonic Self-Expandable NiTi Stent under Peristaltic Movements

**DOI:** 10.1155/2013/370582

**Published:** 2013-06-06

**Authors:** Sergio Puértolas, Eduardo Bajador, José A. Puértolas, Enrique López, Elena Ibarz, Luis Gracia, Antonio Herrera

**Affiliations:** ^1^Department of Mechanical Engineering, University of Zaragoza, Maria de Luna 3, 50018 Zaragoza, Spain; ^2^Department of Gastroenterology, Miguel Servet University Hospital, Paseo Isabel la Católica 1, 50009 Zaragoza, Spain; ^3^Department of Material Science, University of Zaragoza, Maria de Luna 3, 50018 Zaragoza, Spain; ^4^Department of Manufacturing Engineering, University of Zaragoza, Maria de Luna 3, 50018 Zaragoza, Spain; ^5^Department of Surgery, University of Zaragoza, Domingo Miral, 50009 Zaragoza, Spain; ^6^Department of Orthopaedic Surgery and Traumatology, Miguel Servet University Hospital, Paseo Isabel la Católica 1, 50009 Zaragoza, Spain

## Abstract

Managing bowel obstruction produced by colon cancer requires an emergency intervention to patients usually in poor conditions, and it requires creating an intestinal stoma in most cases. Regardless of that the tumor may be resectable, a two-stage surgery is mandatory. To avoid these disadvantages, endoscopic placement of self-expanding stents has been introduced more than 10 years ago, as an alternative to relieve colonic obstruction. It can be used as a bridge to elective single-stage surgery avoiding a stoma or as a definitive palliative solution in patients with irresectable tumor or poor estimated survival. Stents must be capable of exerting an adequate radial pressure on the stenosed wall, keeping in mind that stent must not move or be crushed, guaranteeing an adequate lumen when affected by peristaltic waves. A finite element simulation of bell-shaped nitinol stent functionality has been done. Catheter introduction, releasing at position, and the effect of peristaltic wave were simulated. To check the reliability of the simulation, a clinical experimentation with porcine specimens was carried out. The stent presented a good deployment and flexibility. Stent behavior was excellent, expanding from the very narrow lumen corresponding to the maximum peristaltic pressure to the complete recovery of operative lumen when the pressure disappears.

## 1. Introduction

Colorectal cancer is the second most prevalent cancer in the world with incidence of one million new cases per year and mortality of about 529,000 deaths [1]. In advanced stages, the tumor tends to grow inside the colon lumen, causing stenosis that may block the passage of stool. Obstruction has been reported in 7–29% of patients with colorectal cancer [2]. Patients with malignant large-bowel obstruction tend to have advanced disease and be poor surgical candidates.

The traditional method of managing complete or subtotal cancer colonic obstruction is surgical, but in the emergency setting, surgery carries a high mortality (15–20%) and high morbidity (45–50%) with increased prevalence of intensive care stay, infections, and complications related to stomas [3].

Moreover, even when the tumor is surgically resectable, after the tumor excision, the surgeon should make a temporary colostomy, because a dirty colon cannot be shunted. Therefore, the initial surgery (resection of the primary tumor and colostomy) must be followed by a second intervention to perform the intestinal anastomosis sometime after.

To avoid these disadvantages, endoscopic placement of self-expanding metal stents (SEMS) to relieve colonic obstruction has been introduced more than 10 years ago [4]. If the patient is a poor candidate for surgical resection because of underlying illness or has unresectable or very widespread metastasis, stenting is a widely accepted alternative to surgical intervention (i.e., stenting for palliation). When used for this purpose, an important factor to consider is the duration of stent patency. In a recent multicenter study, there is a stent patency of 96% [5], on the other hand if we compare the results of treatment of obstruction by colostomy or SEMS, these have fewer complications, are less costly, and have shorter hospital stay [6]. If the patient has a surgical resectable tumor, the SEMS is used as “bridge” to elective single-stage surgery, most often without a stoma, thereby significantly reducing the mortality and morbidity [7–10].

Recent technologic advances have seen the advent of stents that expand to greater diameters and have increased flexibility, facilitating placement across tortuous paths and more angulated strictures, like those encountered in the colon [11–13]. Stents must be capable of exerting a radial pressure on the bowel wall stenosed, allowing bolus transit, while keeping good flexibility to adapt well to the walls and prevent migration or obstruction. The peristaltic wave which allows the intestinal transit affects the stent, which must not move or crushed, guaranteeing an adequate lumen. A specific finite element (FE) simulation concerning peristaltic movements affecting the global behavior of the stent would help to know better its response. Therefore this work tries to simulate how the forces generated by intestinal peristalsis affect the stent functionality.

## 2. Materials and Methods

**Figure 1:**
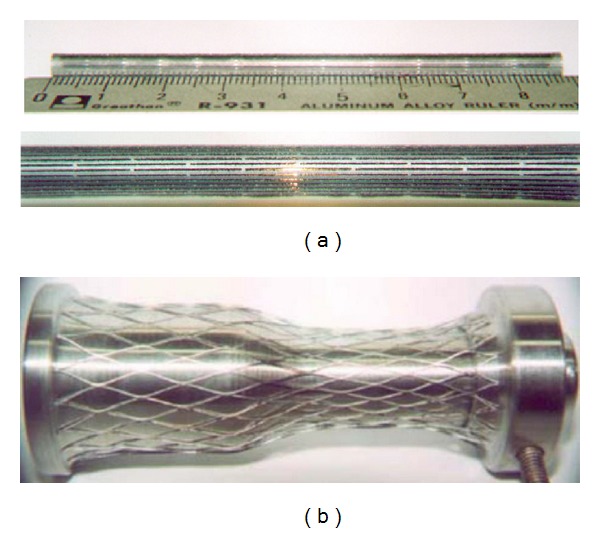
Stent manufacturing: (a) slotted tube, (b) stent after expansion process.

The stent geometry analyzed in this paper corresponds to the prototype proposed by the same authors in [11, 14]. It is a self-expanding stent with diamond cell type and bell-shaped profile with longitudinal variable radial strength. The stent was manufactured in the laboratory of Material Science Department, under authors' supervision, from a NiTi thin-walled tube of 4.5 mm outer diameter with a wall thickness of 400 *μ*m, with a composition of 50.8 at% nickel and 49.2 at% titanium (supplied by Minitubes Ltd, Grenoble, France). A laser-cutting technique was used to obtain a strut width of 400 *μ*m and variable strut length from 6.15 mm to 16 mm (Figure 1(a)). The final bell-shaped stent was obtained by plastic deformation process at cryogenic conditions and posterior thermal annealing treatment [11, 14] (Figure 1(b)). A more detailed explanation about the whole process including thermal treatments can be found in [14] for a preliminary design. The final design reported in this work, with the desired features according clinical requirements, was achieved after several trials. That configuration is denoted as undeformed (zero load state, stress free) configuration.

**Figure 2:**
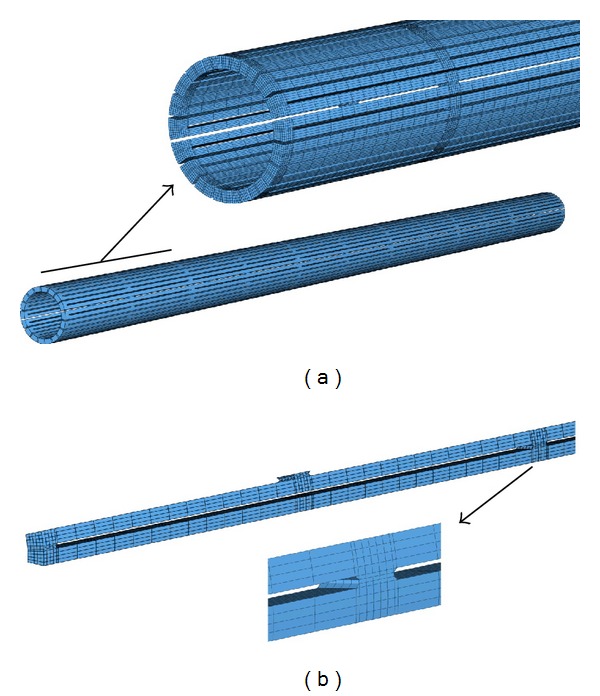
FE model of the slotted tube: (a) global model, (b) detail of knot between struts.

**Figure 3:**
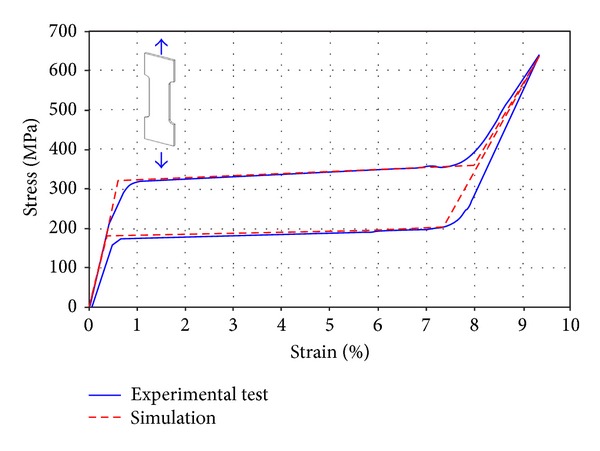
Adjustment of parameters for UMAT subroutine from the results obtained in tensile tests.

**Figure 4:**
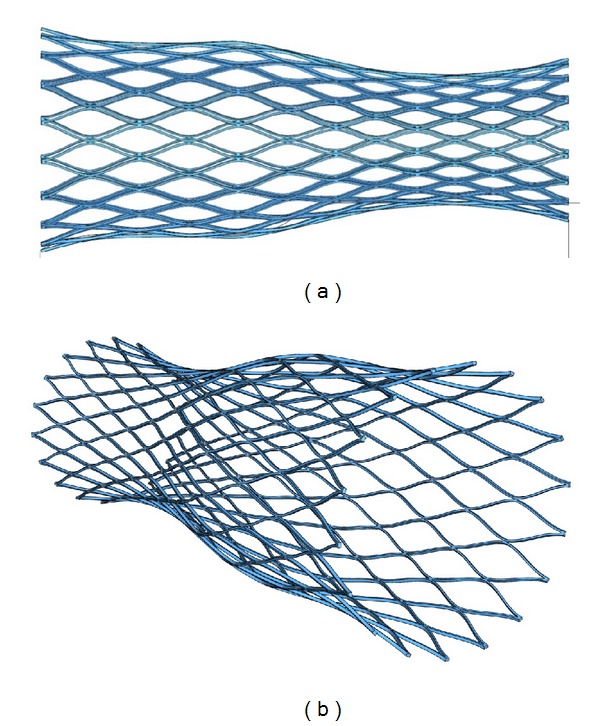
FE model of the expanded stent.

In order to get an accurate geometric model, the previous deformation process was simulated, starting with the slotted tube, expanding and fixing its final shape. In this respect, a 3D finite element model was developed (Figure 2), from a solid 3D CAD model, using brick and wedge linear elements. The total number of elements in the model was 184770 (180450 brick type, 4320 wedge type). The forming process was simulated in the way described in [14], using a rigid surface for the forming tool inserted in the tube. This is a realistic assumption, taking into account that its stiffness is several orders of magnitude upper to stent structure. The geometric model and subsequent meshing were made using the I-Deas software [15]. Contact conditions (frictionless) were imposed between the stent-node-based surface (inner-node set) and the outer surface of the forming tool, and a user-defined material subroutine (UMAT) for the NiTi mechanical behavior, based on the developments included in [16], was employed using Abaqus Standard 6.10.1 software [17], after previously carrying out an adjustment of parameters from the results obtained in tensile tests (Figure 3). Once the final shape was achieved (Figure 4), the stress and strain fields were removed.

Usually, the stent is mounted in a preloaded constrained position on a delivery catheter. When the obstruction has been localized, a guire is passed through the lumen beyond the stenosis and then the stent is advanced over it and positioned across the stricture. Then, the constraining mechanism is released and the expansive forces cause radial opening of the lumen. Then, in order to get a more realistic simulation, both catheter introduction and liberation were simulated in two steps.

- Crimping: the stent was compressed into a 14 Fr catheter (1 Fr = 0.333 mm) defined as a rigid surface. The stent was fixed in axial direction and free on radial direction allowing deforming freely under the contact pressure of a crimping tool and catheter with no contact friction.
- Releasing: while holding the stent fixed in axial direction and slipping the rigid surface that models the delivery system with frictionless contact, the stent was progressively recovering its shape but limited by the compressive force exerted by the colonic tissue.

Since the colon transports solid material, it is necessary to cause its movement through a local contraction of great strength, which is spreading in waves stretches that resemble a worm motion. So, after positioning the stent, the application of a pressure wave on the external surface of the duct-stent structure is modeled to simulate the response to peristaltic movement, which is the most demanding mechanical requirement. The loading conditions consider a wave motion with amplitude which produces a uniform constriction in the section of duct stent that has a diameter at maximum compression of 6 mm, according to clinical experience. A ring compression was modeled as a displacement driven process. That option was choosen after studying different alternatives (displacement wave, pressure wave) and after verifying in clinical experimentation that the radial recovering force of the stent was appropriate when it is reached by the peristaltic wave, suffering the maximum radial compression, being able to achieve a complete recovering when the peristaltic pressure disappears.

Concerning material properties, NiTi has a super elastic behaviour which lends to the property of withstanding large elastical deformations with relatively low tensions. This property is due to the change of phase austenite-martensite-austenite which the material undergoes when it is subjected to tension. The shape of hysteresis curve characteristic of NiTi alloys allows achieving a calibrated behavior of the stent, providing forces adapted to clinical needs to treat each stenosis.

There were several uniaxial tensile tests using a universal testing machine, Instron 5565. Each NiTi test specimen (dog-bone shaped, 60 mm long, 10 mm width, 1 mm thick, obtained from a sheet supplied by the same manufacturer than the tube and with the same composition and properties) was subjected to complete cycle of loading and unloading, making a displacement control. During load cycle the material reaches complete transformation to martensite, getting maximum strain of 8%. Young modulus of the austenite and martensite phases (*E*_A_, *E*_M_), the different values of phase transition stresses (*σ*_*s*_^AM^, *σ*_*f*_^AM^, *σ*_*s*_^MA^, *σ*_*f*_^MA^) for a specific reference temperature (22°C), and maximum strain (*ε*_*L*_) were derived from the experimental tests (Table 1, Figure 3).

**Figure 5:**
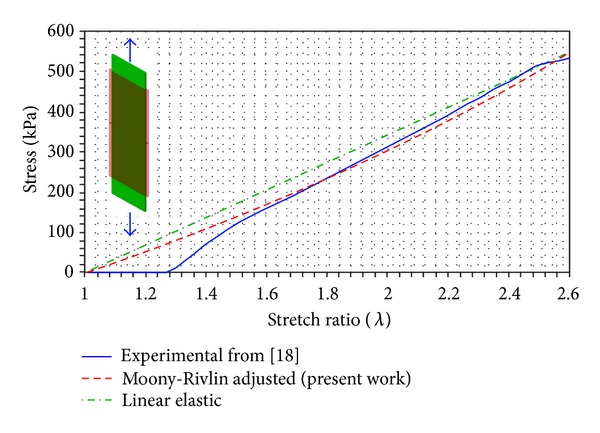
Adjustment of parameters for Mooney-Rivlin hyperelastic model of colonic tissue from the results reported in [18] for large bowel. *C*_10_ = 42.20 MPa; *C*_01_ = 2.11 MPa; *D*_1_ = 0 (correlation coefficient *R* ^2^ = 0.993). Stretch ratio: actual length/initial length.

Colonic tissue has been modeled using a homogeneus, isotropic, and hyperelastic constitutive model. The constitutive law is described in terms of a strain energy density function, taken as a polynomial Mooney-Rivlin form [19]. Only a few studies concerning mechanical behaviour of colonic tissue can be found in the specialized literature [18, 20], so in this work the data from [18] were used (Figure 5). 

Finally, in order to check the reliability of the simulation, a clinical experimentation with porcine specimens was carried out. For stenosis creation, through laparotomy, an autologous peritoneal patch measuring 6 cm in length and 4 cm width was harvested and folded into a double-layer band (6 × 2 cm). The peritoneal band was wrapped around the sigmoid colon approximately 20 cm from the anus. A 6 mm diameter probe was placed inside the colon and the band was then tightened and sutured onto the colon. Finally, a contrast enema was performed to document the presence of stenosis [14]. The colon was simulated as a hyperelastic cylindrical duct with a mesh of 18144 membrane elements with a thickness of 0.9 mm, with a stiffness accounting for colon wall and peritoneal band in the zone where both tissues were superimposed (a stiffer zone corresponding to the induced stenosis).

In order to carry out the stent deployment an introducer sheath was placed inside the colon crossing the stenosis. An adequate catheter was used to place the stent at the exact deployment site, injecting contrast medium for checking the exact position. Once the stent was positioned, the stent was deployed by keeping it in place with the pusher and sliding the introducer sheath backwards. Fluoroscopic images of the stenosis were taken before and after deployment to verify the appropriate deployment [14]. Finally, endoscopic images were taken in different steps during the peristaltic movement.

## 3. Results and Discussion

The expansion process from the slotted tube leads to a bell-shaped stent with diameters of 24.5 mm and 35.0 mm at the respective ends. Those values agree with the actual stent dimensions.

**Figure 6:**
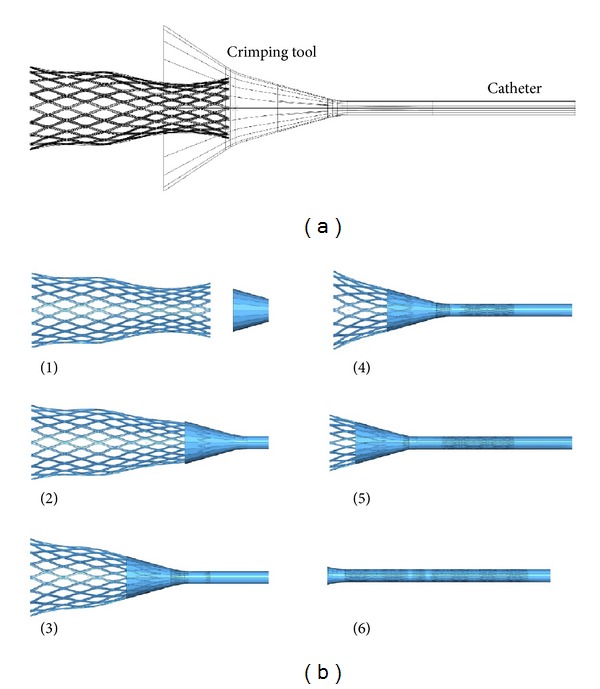
(a) Model of rigid surface used to crimp tool and catheter, (b) simulation of stent introduction into the catheter.

Stent introduction into a 14 Fr catheter was carried out as depicted in Figure 6. The stent achieves a cylindrical form with an outer diameter corresponding to the inner diameter of the catheter. Phase transformation austenite-martensite was detected at the knot, but in any case, the maximum strain reaches the value of *ε*_*L*_. 

**Figure 7:**
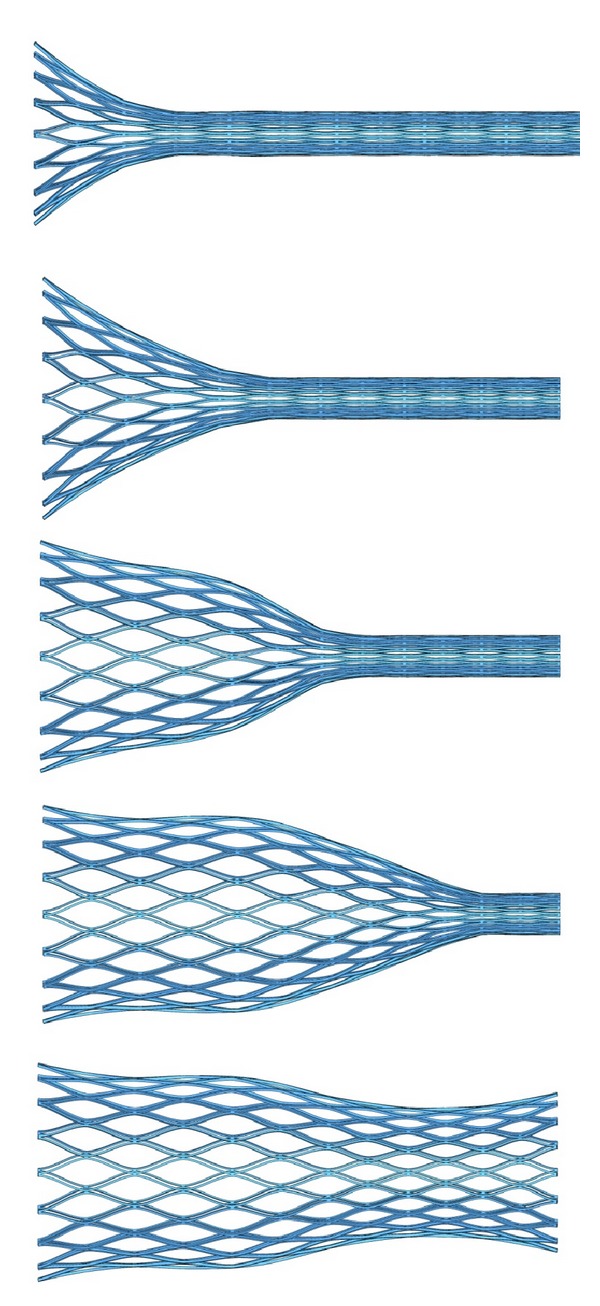
Simulation of stent deployment against the colon wall. Colon wall was removed to see the stent shape.

**Figure 8:**
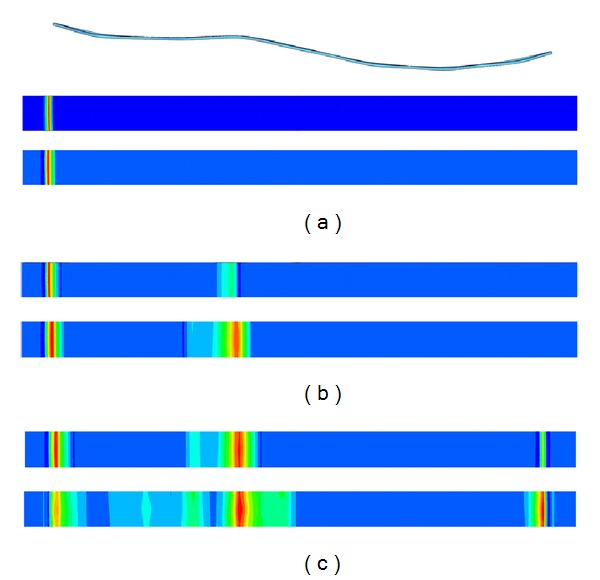
Stresses at the colon wall along deployment process, (a) Stress appearing and growing when distal end is released; (b) stress appearing and growing when intermediate bell-shaped zone is released; and (c) stress appearing and growing when proximal end is released.

Concerning stent deployment, the process is shown in Figure 7. A perfect deployment can be observed with a final shape very close to the initial undeformed configuration and with diameters of 20.5 mm and 27.5 mm at the respective ends. So a diameter decrease of 16.3% and 21.4%, respectively, appears as consequence of colon stiffness. The previous situation implies a compressive force exerted by the stent on the colon wall.  Figure 8 shows the stresses in the colon wall due to that compressive force along the liberation process. It can be observed that as the stent is released from the catheter, a contact between stent and colon walls appears. In the final position, three zones of maximum compression are detected.

When the deployment starts, a first contact zone is detected at the colon proximal end. The deployment progress enables another contact zone correspondent to the medial curvature of the stent. Finally, when the complete deployment is reached, a contact zone appears at the colon distal end. Any case, the stresses in the colon wall never reach dangerous values capable of producing damage in the tissue. 

**Figure 9:**
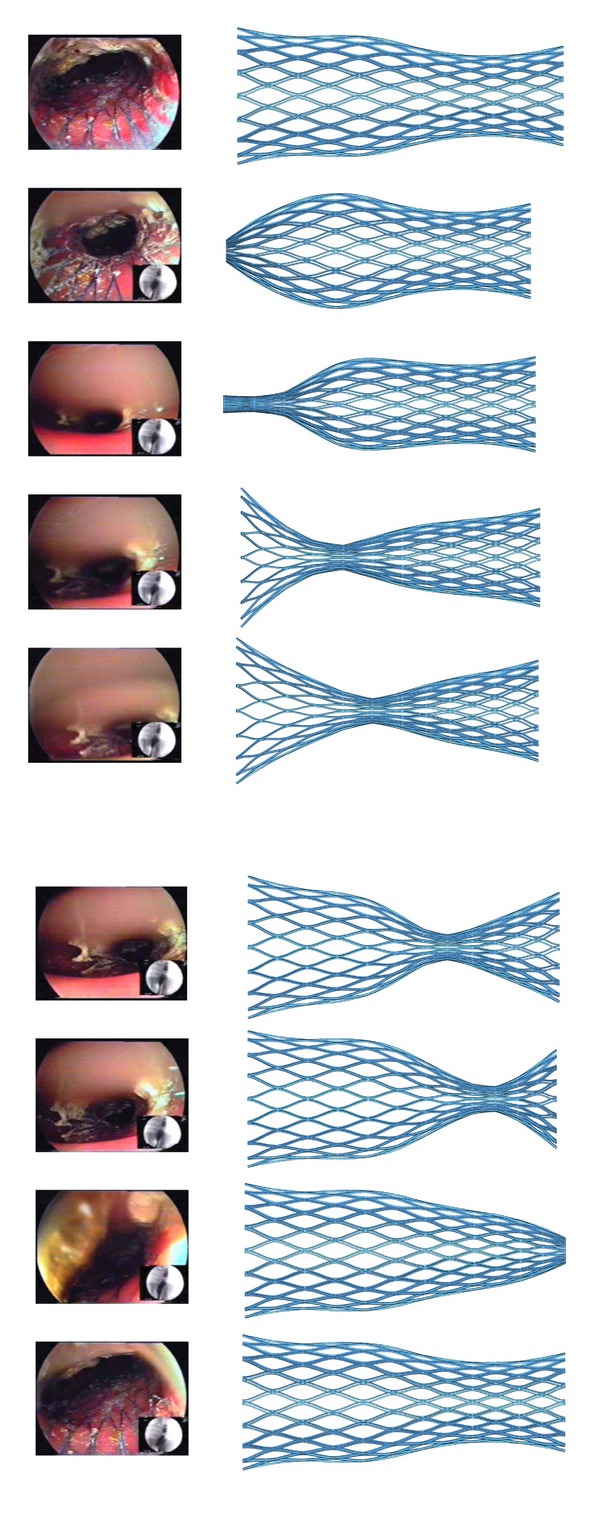
Peristaltic movement. Comparison between FE simulation and endoscopic images.

Finally, for the peristaltic movement simulation, Figure 9 shows a comparison between FE deformed geometries and endoscopic images along the movement sequence. It can be observed how both sequences are coincident and how the stent recovers the shape previous to the peristaltic movement effect and remains in its original position without any displacement along colon, avoiding thus stent migration.

**Figure 10:**
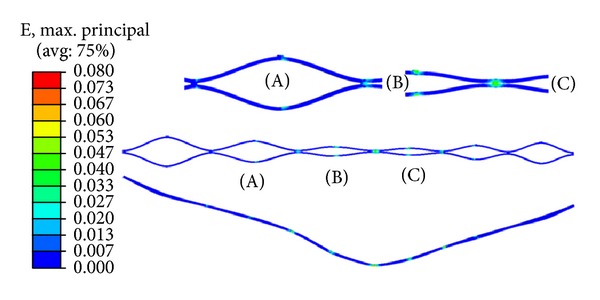
Strain distribution corresponding to the maximum peristaltic compression in the medial zone.

**Figure 11:**
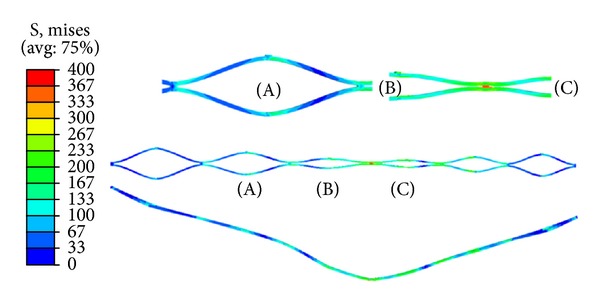
von Mises stress distribution corresponding to the maximum peristaltic compression in the medial zone.

An essential topic concerns the strain and stress values reached in the stent with the maximum peristaltic compression, capable to cause the stent crushing. The strain and stress maps corresponding to the maximum peristaltic compression in the medial zone of the stent are shown in Figures 10 and 11, respectively. Similar maps can be depicted at any step of the peristaltic movement. All the strain values remain under *ε*_*L*_, so no yielding occurs, and the stent is capable of recovering its initial shape and size, so crushing is not a problem.

In the clinical experimentation, the stent presented a good deployment with very good flexibility. The observed diameters corresponding to maximum expansion, measured on fluoroscopic images, were 19.5 mm and 25.5 mm at the respective ends (−4.9% and −7.3% with respect to the FE simulation results). The stent behavior during peristaltic movements was excellent, expanding without difficulty from the very narrow lumen corresponding to the maximum peristaltic pressure till the complete recovery of operative lumen when the pressure disappears (Figure 9).

Advantages of stenting in the colon obstruction before surgical resection include acute restoration of luminal patency with preoperative decompression, clinical stabilization and a proper colon cleansing, and the allowance of time for surgical optimization and staging before resection, so it is possible to perform the surgical intervention in a single stage. Complication rates associated with SEMS range between 14 and 42% [21–25], and most complications are minor. Minor complications include SEMS occlusion due to tumor in growth; this may lead to recurrent colonic obstruction in 10–25% of endoscopically palliated cases [26]. Severe complications occur in up to 10% of patients. These consist of stent migration, perforation, and sepsis. Stent migration rates range between 10 and 12%. Migration can happen a few days after stent deployment or later in the disease course. These were probably a result of tumor shrinkage from adjuvant chemotherapy [27] or to the effect of intestinal peristalsis. A complete study about stent migration and colon perforation can be found in [28].

While stents work well in the vascular structures, biliar track, or ureter, its operation is more difficult in the colon, because of the special peristalsis and fecal content characteristics, favoring its displacement or obstruction. The large intestine presents a complex peristalsis, consisting of deep circular and circular muscle contractions, causing a wave which progresses longitudinally along the intestine and leads to feces until the rectal ampoule. 

Finite element simulation of NiTi colorectal stent has been carried out for a better understanding of the mechanical behaviour of a bell-shaped stent under peristaltic movements. It is a self-expanding stent with diamond cell type and bell-shaped profile with longitudinal variable radial strength getting a better resistance to the migration. The simulation results provide a good structural response to external pressure waves used to model the peristaltic movements.

In validating colonic stent, we found that finite element analysis reproduces the stent compression-deployment process adequately by comparison with clinical experimentation. The idealized cylindrical straight geometry assumed to model colonic duct wall makes possible the application of peristaltic reflex and has a good approximation of the mechanical response of the stent structure. The modeled peristaltic pressure wave is in accordance with [29, 30]. Clearly, patient-specific colonic duct exhibits wide variations in terms of geometry, size, material properties, and loading conditions. These simplifying assumptions somewhat limit the present results; nevertheless, the analysis introduces a new approach to evaluate the mechanical response of stents under peristaltic movements. The analyzed stent has proved to be adequate to prevent migration and crushing.

The other simulated processes (expansion, introduction into the catheter and deployment) have demonstrated the ability of the analyzed stent for supporting every step in the clinical process required to the stent implantation, and hence the self-expanding stent with diamond cell type and bell-shaped profile with longitudinal variable radial strength is a design reliable for colonic applications.

An essential point in the simulation is the approach used for the colonic tissue. In the specialized literature, several works about mechanical behaviour of soft tissue in coronary arteries [31, 32] or esophagus [33] can be found, but there are only a few studies concerning mechanical behaviour of colonic tissue [18, 20]. For future improvements, in [18] experimental data for colonic tissue are included; those data could be used to adjust an accurate constitutive model.

Another important topic concerns fatigue behaviour of NiTi alloy. In this work, only one peristaltic pressure cycle has been simulated, but the peristaltic movements produce continuous cycling loads. Moreover, the cycling loads vary from zero to the maximum amplitude, corresponding to the maximum expansion rate in the stent. Cycling loads would cause stent breaks by cumulative damage and fatigue. However, the frequency of peristaltic movements is less than the corresponding to coronary stents [34].

NiTi alloys fatigue behaviour has been studied for applications with higher number of cycles than the corresponding to gastrointestinal ducts with good results [35] but with less cycle amplitudes. However other works are close to the present problem; so in [36], the approach of Coffin-Manson is applied to determine the fatigue life in NiTi medical devices, and in [37, 38], experimental results for NiTi low-cycle fatigue life are presented. In both cases (high-cycle, and low cycle fatigue life), the ability of NiTi alloy to undergo cycling loads causing fatigue is demonstrated, so the stent would be able to undergo continuous cycling loads produced by the peristaltic movements.

## 4. Conclusions

A finite element simulation and a clinical experimentation of a bell-shaped nitinol stent functionality have been done. Every step in the implantation process was simulated (catheter introduction, releasing at position, and the effect of peristaltic wave). The stent presented a good deployment and flexibility, both in the simulation and in the clinical experimentation. The stent behavior during peristaltic movements was excellent, expanding without difficulty from the very narrow lumen corresponding to the maximum peristaltic pressure till the complete recovery of operative lumen when the pressure disappears.

The results of the study show that Niti self-expanding bell-shaped stent is as an effective alternative to relieve colonic obstruction, either as a bridge to elective single-stage surgery avoiding a stoma or as a definitive palliative solution in patients with irresectable tumor or poor estimated survival. 

## Figures and Tables

**Table 1:** NiTi material parameters which define its stress-strain curve. UMAT input material parameters for constitutive model implemented in Abaqus.

Parameter	Description	Value
*E*_A_	Austenite elasticity modulus	52650 MPa
*ν*_A_	Austenite Poisson's ratio	0.33
*E*_M_	Martensite elasticity modulus	38250 MPa
*ν*_M_	Martensite Poisson's ratio	0.33
*ε*_L_	Maximum transformation strain	8%
*σ*_*s*_^AM^	Start of transformation austenite-martensite stress	320 MPa
*σ*_*f*_^AM^	End of transformation austenite-martensite stress	360 MPa
*σ*_*s*_^MA^	Start of transformation martensite-austenite stress	200 MPa
*σ*_*f*_^MA^	End of transformation martensite-austenite stress	180 MPa
*T*_0_	Reference temperature	22°C
*C* ^AM^	∂*σ*_*s*,*f*_^AM^/∂*T*	6.7 MPa/°C
*C* ^MA^	∂*σ*_*s*,*f*_^MA^/∂*T*	6.7 MPa/°C
